# A Systemic Review of Available Low-Cost EEG Headsets Used for Drowsiness Detection

**DOI:** 10.3389/fninf.2020.553352

**Published:** 2020-10-15

**Authors:** John LaRocco, Minh Dong Le, Dong-Guk Paeng

**Affiliations:** Ocean Systems Engineering, Jeju National University, Jeju City, South Korea

**Keywords:** electroencephalography (EEG), drowsiness detection, low-cost, consumer EEG, fatigue detection, device portability

## Abstract

Drowsiness is a leading cause of traffic and industrial accidents, costing lives and productivity. Electroencephalography (EEG) signals can reflect awareness and attentiveness, and low-cost consumer EEG headsets are available on the market. The use of these devices as drowsiness detectors could increase the accessibility of safety and productivity-enhancing devices for small businesses and developing countries. We conducted a systemic review of currently available, low-cost, consumer EEG-based drowsiness detection systems. We sought to determine whether consumer EEG headsets could be reliably utilized as rudimentary drowsiness detection systems. We included documented cases describing successful drowsiness detection using consumer EEG-based devices, including the Neurosky MindWave, InteraXon Muse, Emotiv Epoc, Emotiv Insight, and OpenBCI. Of 46 relevant studies, ~27 reported an accuracy score. The lowest of these was the Neurosky Mindwave, with a minimum of 31%. The second lowest accuracy reported was 79.4% with an OpenBCI study. In many cases, algorithmic optimization remains necessary. Different methods for accuracy calculation, system calibration, and different definitions of drowsiness made direct comparisons problematic. However, even basic features, such as the power spectra of EEG bands, were able to consistently detect drowsiness. Each specific device has its own capabilities, tradeoffs, and limitations. Widely used spectral features can achieve successful drowsiness detection, even with low-cost consumer devices; however, reliability issues must still be addressed in an occupational context.

## Introduction

Drowsiness is defined as the transition between the states of responsiveness and sleep, during which reaction times are reduced (US Dot National Highway Traffic Safety Administration, 2018). Drowsiness or fatigue is a major cause of road accidents and has significant implications for road safety, due to clear declines in attention, the recognition of dangerous drivers, and the diminished vehicle-handling abilities associated with drowsiness (Wang, 2011; Solaz et al., 2016). In addition, drowsiness-related accidents cost billions of US dollars and result in the loss of lives in industry, including transportation, manufacturing, mining, maritime, and aerospace sectors. Thus, developing a reliable, non-invasive method for drowsiness detection can save both money and lives (US Dot National Highway Traffic Safety Administration, 2018).

Key economic sectors, such as transportation, construction, security, and manufacturing, reported loss of productivity and lives due to drowsiness (Wang, 2011; Solaz et al., 2016). In the transportation sector, drowsiness-influenced road accidents represent social and economic problems worldwide. In the European Union (EU), 25% of road accidents have been associated with fatigue and drowsiness, compared with 40% of fatal accidents in the United States (US) (Solaz et al., 2016; Wei et al., 2018). According to a National Highway Traffic Safety Administration (NHTSA) report, ~83,000 road accidents reported annually in the US are caused by driver fatigue. Their analysis showed that ~416,000 crashes were caused by drowsy driving during the 5-year period from 2005 to 2009 (Wang et al., 2017; US Dot National Highway Traffic Safety Administration, 2018). In 2017, the NHTSA report reported that 3,166 fatalities resulted from distraction-affected crashes (US Dot National Highway Traffic Safety Administration, 2018). The factors that contributed to drowsiness included long working hours, the use of medication, lack of sleep, and continuous driving (Zhang et al., 2017). However, the exact definition of drowsiness is highly variable.

The term drowsiness is sometimes used interchangeably with the term fatigue in the literature. Although physiological state detection has been used to detect either (or both) states, researchers have defined certain differences between drowsiness and fatigue. Fatigue was defined as the decrease in physical and mental performance resulting from exhaustion (Vuckovic et al., 2002; Cabrall et al., 2016). Drowsiness can be a symptom of fatigue, which can occur without drowsiness (Vuckovic et al., 2002). Other concepts, such as microsleep, can be used to describe a similar lack of responsiveness but have mechanisms distinct from those associated with fatigue and drowsiness (Davidson et al., 2007; Izquierdo-Reyes et al., 2016). Electroencephalography (EEG) has been used to identify these mechanisms, but the body of such work examining these other concepts is less well-defined than the total body of work associated with drowsiness research (Bryan Van Hal and Bossemeyer, 2014; Cabrall et al., 2016; Wang et al., 2017; Rundo et al., 2019).

The prediction of drowsiness using EEG is a well-defined research topic. Approaches that utilize conventional EEG systems have advantages for the quantitative assessment of alertness levels, which requires expensive computational signal processing (Mard et al., 2011; Correa et al., 2014; Shabani et al., 2016; Zhang et al., 2017). Observing changes in the power spectra or spatio-temporal features of EEG frequency bands have commonly been used to detect subject drowsiness, but other methods have been investigated (Ayala Meza, 2017; Min et al., 2017; Majkowski et al., 2018). EEG-based drowsiness detection systems could be easily integrated into protective or occupational headgear for use in occupations that require such equipment (Wilaiprasitporn and Yagi, 2016).

Research- and medical-grade EEG systems rely on the use of dozens of channels, rendering such systems impractical for real-world occupational use (Ries et al., 2014). In contrast, low-cost EEG systems offer potential solutions for drowsiness prediction. These systems typically include fewer electrodes than medical and research headsets, but their low prices make them accessible to hobbyists, small businesses, and developing countries. The use of consumer EEG headsets as drowsiness detectors has been previously investigated (Rodríguez et al., 2013; Van Hal et al., 2014; Salehi et al., 2015). A review of consumer EEG headsets as research tools was investigated, but it did not include occupational contexts (Sawangjai et al., 2019).

This review was conducted to evaluate the feasibility, complexity, and difficulty of using low-cost EEG systems for occupational drowsiness detection, such as drivers and security guards. PRISMA standards for systematic reviews were considered (Moher et al., 2009). The initial problem was the cost of drowsiness on economic productivity and safety. The implementation of low-cost, EEG-based detection could make the technology more accessible. Drowsiness detection systems implemented with low-cost EEG devices were compared. The successful outcomes were low-cost, robust implementations. A validation required study designs replicating occupational conditions with multiple subjects. A systematic search was conducted investigate prior implementations of low-cost EEG-based drowsiness detection systems.

## Search Methodology

### Summary

In recent years, the number of portable, low-cost EEG-based systems available on the market has increased (Wei et al., 2018). Research examining the use of low-cost EEG systems has focused on the continuous recording of EEG data and/or the replication of larger EEG analytical systems using portable devices. In this review, we surveyed research papers that described the use of low-cost EEG devices, focusing on the devices where the headset was below $1,000 USD in price, independent of licensing fees: the InteraXon Muse, the Neurosky MindWave, the Emotiv Epoc, the Emotiv Insight, and the OpenBCI. These devices represent a sample of widely-used commercial models. Although other devices and suppliers have been used (Li and Chung, 2015), the search was focused on those non-invasive EEG devices that were below $1000, not marketed as medical devices, accessible to consumers, prominent in the hobbyist community, and have provided tools or options for brain-computer interface (BCI) applications. Table 1 presents a comparison of these commercial, low-cost EEG headsets. Most low-cost headsets use dry electrodes, which are more convenient for casual users. Similarly, most headsets come bundled with software that includes research tools, open-source software, and additional hardware (Lin et al., 2014; Farnsworth, 2017).

**Table 1:** Comparison of consumer EEG headsets.

**Device**	**Electrodes**	**Sampling Rate**	**External Information**	**References**
InteraXon	- Rigid electrode placement	− 256 Hz	- Research Tools for Windows, Mac, and Linux	Doudou et al., 2018
Muse v1, v2	− 4 channels: AF7, AF8, TP9, TP10	− 12 bits	- Source Developer Kit (SDK) for Android, IOS, Windows	
			- Cost: $200 USD	
Neurosky MindWave	- Rigid electrode placement	− 512 Hz	- SDK Available	Doudou et al., 2018
	− 1 channel: AFz	− 12 bits	- Cost: $99.99 USD	
OpenBCI	- Up to 16 channels	− 256 Hz	- Open-source software, firmware, and hardware	Doudou et al., 2018
	- Flexible electrode placement at 35 locations	− 24 bits	- Cost: $500 USD for 8 channels, $949 USD for 16	
Emotiv Epoc, Flex, and Insight	- Rigid electrode placement	− 128 Hz	- Research Tools for Windows, Mac, and Linux	Doudou et al., 2018
	- Epoc: 14 channels (AF3, F7, F3, FC5, T7, P7, O1, O2, P8, T8, FC6, F4, F8, AF4)	− 14 bits	- Cost: $799 USD (Epoc), $299 USD (Insight)	
	- Insight: 5 channels (AF3, AF4, T7, T8, Pz)			

### Headset Information

The primary investigated headsets were the InteraXon Muse, the Neurosky MindWave, OpenBCI, and the Emotiv Epoc and Insight.

#### InterAxon Muse

The InteraXon Muse is a compact EEG system that measures brain activity via 4 EEG sensors (Muse, InteraXon) and can utilize Bluetooth to send data to nearby devices. Muse claimed that the headband could assist the user to achieve a state of deep relaxation. Based on the 10–20 International electrode placement convention, the dry electrodes were located at FPz, AF7, AF8, TP9, and TP10 (Krigolson et al., 2017). Electrode FPz was utilized as the reference electrode. The specifications detailed correspond to the original Muse device.

#### Neurosky Mindwave

Neurosky developed the single-channel MindWave as a low-cost, single-channel, dry EEG headset that is able to wirelessly transmit EEG via Bluetooth Low Energy or classic Bluetooth (Doudou et al., 2018). The MindWave device consists of a headset, with a T-shaped headband, a wider ear clip, and a flexible arm. The device's reference and ground electrodes are placed on the ear, while the EEG electrode is positioned on the forehead above the eye. Neurosky EEG headsets come with training software, educational apps, and software developer information. Free developer tools are also available for researchers. While Neurosky makes other models, the MindWave was the most frequently used model in the relevant studies (Lin et al., 2014; Doudou et al., 2018).

#### OpenBCI

The OpenBCI Ultracortex Mark IV is an open-source, 3D-printable headset intended to work with any OpenBCI board. It is capable of recording research-grade brain activity EEG. The Ultracortex Mark IV headset is capable of sampling up to 16 channels of EEG from up to 35 different locations, based on the 10–20 International System (Mohamed et al., 2018b). The OpenBCI boards include options for 4 channels, 8 channels, and 16 channels. The OpenBCI is an open-source assemblage of parts, requiring assembly prior to use (Murphy and Russomanno, 2016). Therefore, it is not as widely used as readily-purchased consumer devices, but it theoretically allows greater customization. It has previously been used for drowsiness detection in a driving simulator.

#### Emotiv Insight and Epoc

Emotiv offers both the smaller, cheaper Insight and the larger, more expensive Epoc (and its upgraded counterpart, the Epoc+). The Emotiv Epoc is the most expensive of the investigated EEG headsets, containing more electrodes than the others (de Lissa et al., 2015). It has two electrode arms, each containing sensor electrodes and two reference electrodes. The locations provide coverage of the temporal, parietal, and occipital lobes. Emotiv provides a free companion app for users to monitor their emotions. They also offer pay-to-download games, such as Arena, which allows users to experience mental commands. Emotiv provides a two-tiered SDK for the Epoc. The headset has been used in research, from BCI to brain state detection (Badcock et al., 2013, 2015; Manolova et al., 2016). However, the Epoc and Epoc+ were the most common models found. Results returned using “Insight” as a keyword instead yielded results referencing the Epoc and Epoc+.

### Scope

The purpose of this review is to identify examples and reports that described the successful use of specific, low-cost, consumer EEG headsets for drowsiness detection. These headsets will be referred to as “low-cost” for simplicity for the remainder of this paper. The scope and aims of the review process were not designed to comment on the algorithms and approaches used for drowsiness detection. Even single-channel EEG headsets, including custom-made headsets, have been successfully used for drowsiness detection in a research context (Ogino, 2018). For this review, a successful study was defined as a system that achieved greater than random accuracy in detecting drowsiness using EEG. Simple, robust algorithms for both drowsiness detection and general EEG processing were preferred, as these are likely to be more easily implemented by resource-constrained small businesses, individuals in developing countries, and others who are unable to afford more complex EEG headsets or purpose-built systems. To facilitate comparisons and ensure search replicability, the PRISMA convention on systematic reviews and meta-analyses was followed (Moher et al., 2009). The PRISMA conventional facilitates the process and replication of research reviews. A prior review focused on the broader viability of low-cost EEG as research tools, but not narrower occupational contexts (Sawangjai et al., 2019). Thus, the primary aim of this review was to determine whether commercial, low-cost EEG headsets can easily be used for occupational drowsiness detection.

### Eligibility Criteria

This review focused primarily studies related to low-cost EEG headsets that those non-invasive EEG devices that were below $1,000 USD, not marketed as medical devices, accessible to consumers, prominent in the hobbyist community, and have provided tools or options for brain-computer interface (BCI) applications. Because commercial EEG systems have been publicly available for approximately one decade prior to the current date, only papers published within this period were included, starting from 2009. This year was shortly before the commercial release of the Emotiv, the earliest of the listed systems. Similarly, the total body of relevant results was thought to represent only a fraction of the total work on drowsiness detection; therefore, conference papers, completed dissertations, and validation studies were included. However, conference papers were excluded if they were published within 1 year of a journal paper on the same topic and by the same authors.

### Data Combination

Two independent searchers, co-authors M. and J., gathered their findings in a reference document. Redundant results were eliminated, and information on each study was gathered. Data items included the authorship and publication of each work, the study design, experimental implementation, reported results, and concluding analysis. Specific preprocessing, feature extraction, and classification techniques were recorded, as was any statistical analysis performed on the results. Information on the computing platform used was also recorded. The primary information sought by each searcher included the work's criteria of “success,” “accuracy,” and other performance metrics. Numerical values, such as the confusion matrix, were used to calculate statistical measures, if provided by the work in question.

### Search Strategy and Parameters

Google Scholar, IEEE Xplore, and PubMed were used as primary sources, due to the large databases available using these sources and their prior use in other reviews. All three search resources have been used in prior literature reviews in the field of biomedical engineering; however, many of the results could be accessed by multiple search engines. The resulting papers were grouped according to the model of EEG headset used. Duplicate results were removed by software.

The use of low-cost EEG headsets for drowsiness detection was described by only a limited number of studies, as the relevant results were those that utilized a low-cost EEG headset as the primary EEG recording system. The search included three phases. The first was the search for keywords, which included the terms: electroencephalography (EEG), drowsiness, and the device name. Second, the three keywords were joined by “AND.” During the third phrase of the search, specific words were sought in the title: (encephalography OR EEG) AND (drowsiness OR fatigue OR tired) AND ([*device name*]). The filter words included: drowsiness, fatigue, and tired.

Results from each source were combined, and duplicates were removed. These “filter words” were selected based on their use in prior papers and literature reviews (Vuckovic et al., 2002; Cabrall et al., 2016; Guo et al., 2017; Min et al., 2017). Similarly, any paper that did not include any of the filter words in the title was eliminated.

**Figure 1:**
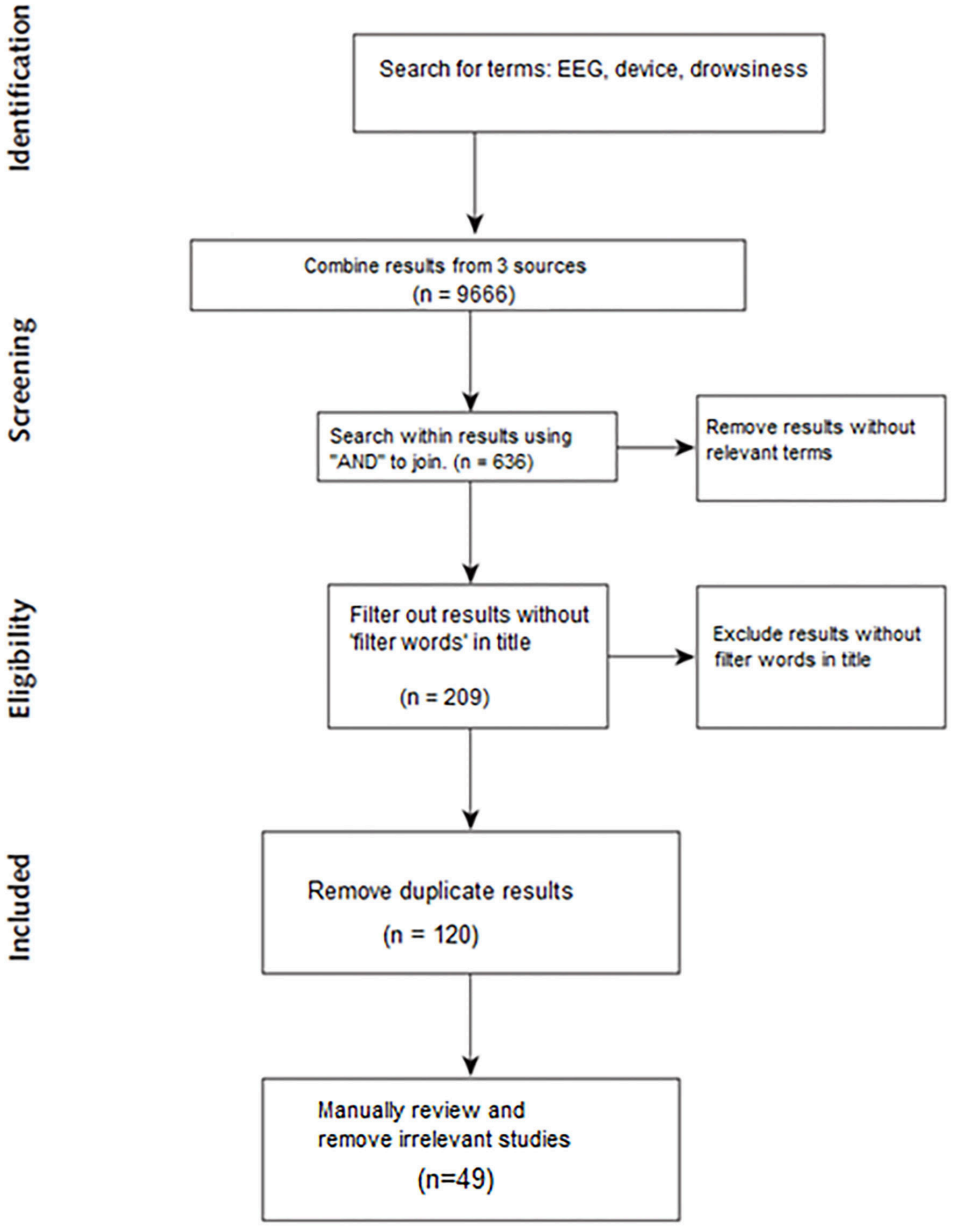
Review search process and winnowing.

The remaining papers were included in the review. The removal of papers through the search process is depicted in Figure 1.

Table 2 summarizes the final results, according to the specific brand of EEG device used.

**Table 2:** Relevant results after search process.

**Brand**	**Papers**
InterAxon	11
Neurosky	16
OpenBCI	5
Emotiv	17

The final papers are further detailed in search results. However, potential bias and limitations had to be accounted for.

### Bias and Error Sources

Possible sources of error included the ranking algorithms used by the search and indexing processes of Google Scholar, PubMed, and IEEE Xplore. The ranking processes of each search engine potentially missed relevant material. The primary purpose of this review was to identify examples describing the use of each consumer EEG headset for drowsiness detection, rather than performing a model-specific critique of each and every device. Similarly, the primary biases in published works would be toward positive results, potentially limiting insights from less successful studies.

A less clear topic was managing potentially relevant studies in affective computing and emotion recognition. Drowsiness has a range of definitions in the research literature, and a range of nearly synonymous terms used interchangeably in different contexts. There was the potential of drowsiness being one of several discrete states detected in an emotion recognition study, rather than an exclusively binary classification (Tan, 2012). Similarly, other studies integrated other signals than EEG (Polosky et al., 2017). In such cases, each study's structure was evaluated to determine if the system, as reported, could be used as to estimate drowsiness. If not, it was excluded from the final review (Alchalabi et al., 2018).

### Data Management

Each search result would be evaluated for relevant data items. These include system parameters and study parameters. System parameters are those relevant to the drowsiness detection system, including the algorithms used for feature extraction and classification. Study parameters include those relevant to the entire study, including experimental design, cohort size, and performance results. The reported classifier accuracy, how reliably a device rates drowsiness, was the primary performance result.

## Results

### Overview

The use of low-cost EEG headsets represents a logical progression based on the accomplishments reported using clinical and research-grade systems. These tasks range from brain-computer interfaces (BCIs) to brain-state rating to drowsiness detection. Much of the reported work has been context-specific, necessitating a thorough look at each study, and some works listed multiple headsets or lacked an experimental component, requiring that they be listed separately. The experimental use of each headset is described.

Crowley et al. (2010) performed psychological tests to induce stress and correlated the results with measured attention and meditation signals, using a Neurosky Mindset. They were able to detect when a subject's emotions changed using the Stroop and Towers of Hanoi tests. Both tests used in this study resulted in clear indications of subject stress and alertness changes, based on the attention and meditation parameters measured by the headset. However, this study would be cited as the basis for others.

Wei (2017) wrote a doctoral dissertation on drowsiness detection based on the detection of neural activity. The document largely described the real-life challenges faced by drowsiness detection in the context of BCI. In addition, experimental work on calibration was performed. Despite the research relevancy, a research 32-channel Quik-Cap Neuroscan EEG headset was used.

Wei et al. (2018) described the potential advantages of smaller, non-hair bearing (NHB) dry electrode headsets relative to larger ones. Smaller headsets that did not offer full coverage of the head were less affected by hair than larger ones. Specific devices named included the Neurosky and InterAxon devices. Potential advantages, including cost and ease of occupational use, were discussed.

Doudou et al. (2018) listed a number of consumer and portable EEG headsets (including all of the headsets reviewed here) and rated them using a number of parameters. Their particular focus was driver-based drowsiness detection. However, the authors did not perform any direct, experimental comparisons. The complete omission of any results was explicitly mentioned in their future work sections, however.

Lakhan et al. (2019) used consumer headsets in affective computing. With a study of 200 healthy subjects, they claimed predictive accuracies approximating those on costlier EEG systems. They used an OpenBCI EEG system for their work. The separate tasks included affective video selection and emotion recognition. No other low-cost headsets were investigated for the study, although they were mentioned in the article. The article lacked extensive discussion of potential context-specific specific advantages or limitations.

Majumder et al. (2019) performed a review of drowsiness detection. The review covered both consumer EEG devices and more purpose-built devices. It was found that power spectral densities of EEG bands were the most commonly utilized features across studies. The final conclusion was that identifying the specific EEG bands and brain sites would limit the need for EEG electrodes, reduce processing requirements, and improve accuracy.

Wexler and Thibault (2019) took a critical view of consumer EEG headsets and many of the claims made regarding their use. In particular, the authors reported that such consumer devices could serve as drowsiness detectors, despite a lack of reliability with regards to the identification of other brain states. They also discussed the legal and ethical complexities of such devices. Many issues were raised, but not all fully addressed.

### InteraXon Muse

Bashivan et al. (2015) collected EEG data from 16 individuals. The authors used support vector machines (SVMs), sparse logistic regression, and deep belief networks (DBN) to discriminate among states of mind induced by different video inputs. The results demonstrated the significant potential for wearable, consumer EEG devices to differentiate among different cognitive states in different situations.

Krigolson et al. (2017) used a Muse for their BCI research. The authors used *t*-tests to observe and quantify statistically significant differences in event-related potentials in 60 subjects, including the N200 and the P300, during both a visual oddball task and a reward-learning task. Statistical tests were conducted for each case.

Rohit et al. (2017) used a Muse for real-time drowsiness detection. Spectral features were used with an SVM classifier on a total of 23 subjects. The study also investigated a blink-based method of drowsiness detection but found this method to be less accurate than the spectral power-based method.

Almogbel et al. (2018) investigated a single subject in a simulated driving task. Temporal feature vectors, from each of the Muse headset's 4 channels, were fed into a convolutional neural network (CNN). Various cognitive workloads were compared in both urban and rural driving scenarios. The CNN was used to estimate the workload based on EEG. The highest accuracy across scenarios was 95.3%. However, no field testing was conducted.

Bakshi (2018) detailed a system to detect cognitive workload through EEG. A Muse headband was used to collect EEG from 28 subjects, and spectral features were calculated for each band. For classification, a linear SVM, a radial basis SVM, a logistic regression model, and a shallow artificial network were used. The linear SVM was easily able to achieve an average accuracy of 99.1%. However, the system was not validated in live trials.

Teo and Chia (2018) proposed using EEG to detect interest and monotony while subjects were immersed in a virtual reality (VR) simulation. Users were exposed to a VR roller-coaster experience while wearing an EEG headset. Using a deep learning approach, accuracy rates of 78–96% were achieved. While “detecting interest” was a novel concept, more supporting research could have been cited.

Araújo (2019) used a second version Muse for drowsy driver detection in a thesis. A scaled artifact rejection was implemented based on the measured spectral power, prior to bandpass filtering. The features used were the power spectral density of different EEG bands. An artificial neural network was used for classification. Three subjects were used for model generation, training, and testing. Final testing accuracies included 70.8, 75.8, and 96.7% for a total average of 81.1% on testing data. In addition to the small sample size, the age of subjects was not addressed.

Foong et al. (2019) used EEG band-based power spectra to identify drowsiness in 29 subjects. Decreases in alpha and beta band power and increases in the theta band power were cited as signature features of drowisiness detection. The final reported accuracy was 93.8 ± 8.2%. A negative-unlearned (NU) algorithim, consisting of a combination of SVM and radial basis function (RBF) was positively reported on. However, the offline implementation precluded assessments for real-time performance.

Mehreen et al. (2019) used multimodal signals from a Muse headset for drowsiness detection. In addition to EEG power spectral features and blink detection, they used accelerometer and gyroscope data to detect head movements, with head nodding corresponding to drowsiness periods. They reported a 92% accuracy, using leave-one-out cross-validation with 50 subjects.

Dunbar et al. (2020) simulated a driving task with 25 subjects. A Muse was used in a driving task. Spectral band power was used for the automated classification. Unlike algorithm-based papers, the purpose was to investigate if self-reported measures were consistent with documented electrophysiological changes. The electrophysiological changes and self-reported measures were consistent across subjects. However, a larger population subject size would be required for decisive confirmation.

Hoffmann (2020) combined gamification with EEG-based drowsiness detection. The dissertation consisted of an evaluation of a Muse headset, a companion app, and a larger study. Alpha and beta band power were the main feature used, calculated after filtering. A total of 19 subjects were used in validating the EEG headset. A combination of the EEG headset and app using self-reported measures were used in the larger studies. Analysis of variance (ANOVA) was used for a comparison of EEG across different states. A limitation was studying the app's effect on stress outside of the evaluated metrics, as well as the relatively low size.

Of the entries reporting accuracy, the minimum was 83.3%, and the maximum was 99.1%. As shown in Table 3, these results suggested that the InteraXon Muse could be sufficiently reliable for use as a drowsiness detection system, due to both its convenience and its successful use during physiological state detection.

**Table 3:** InterAxon Muse Studies.

**Paper**	**Year**	**Features**	**Classification**	**Accuracy**	**Size**
Bashivan et al.	2015	Spectral Features	SVM, Regression, DBN	N/A	16
Krigolson et al.	2017	Amplitude	*t*-test	N/A	60
Rohit et al.	2017	Spectral Features	SVM	87%	23
Almogbel et al.,	2018	Raw EEG	CNN	95.30%	1
Bakshi	2018	Spectral Features	SVM, Regression, NN	99.10%	28
Teo and Chia	2018	Spectral Features	Deep NN	96%	24
Araújo	2019	Spectral Features	NN	81.10%	3
Foong et al.	2019	Spectral Features	NU (RBF+SVM)	93.80%	29
Mehreen et al.	2019	Spectral Features, Gyro	Linear SVM	92%	50
Dunbar et al.	2020	Spectral Features	N/A	N/A	25
Hoffman	2020	Spectral Features	ANOVA	N/A	19

### Neurosky MindWave

Jones and Schwartz (2010) wrote a short article reviewing several low-cost EEG devices, including a Neurosky device, and their abilities to detect drowsiness. The signal frequency content was divided into the following clinically relevant frequency bands: alpha (8–13 Hz), beta (14–30 Hz), and theta (4–7 Hz) waves. When comparing the power spectra, the alpha and beta waves decreased when drowsy, while the theta waves remained constant.

AlZu'bi et al. (2013) reported on three feature extraction methods using EEG: power spectral density, log variance, and statistical features. These features were fused into a single fatigue index; however, no accuracy scores were reported.

Shin et al. (2013) used EEG signals combined with an SVM classifier. A total of 5 subjects wore the MindWave for 3 h each night, to capture the onset of sleep and drowsiness. Analysis of variance (ANOVA) was performed on the extracted features, identifying statistically significant (*p* < 0.001) differences between the alert and the drowsiness states. The results reported an accuracy of 88.9% from a single subject, preventing larger validation of the system.

Lim et al. (2014) examined changes in the low alpha EEG band during eye closure. A total of 50 subjects were rated, with periods denoted by the Karolinska scale. The system had a lower accuracy rate than comparable systems, with an accuracy of 31% per second. However, the reported false alarm rate was 0.5%. Comparable MindWave-based systems reported accuracy higher than the reported rate, such as Suprihadi and Karyono (accuracy of 68.11%).

Suprihadi and Karyono (2014) used a MindWave device as a drowsiness detection system. An alarm was triggered when the classifier detected a drowsy state, based on low alpha, high alpha, and theta spectral features. They reported an accuracy of 68.11%.

Abdel-Rahman et al. (2015) designed a mobile app to work with the MindWave EEG headset. They reported a 98% accuracy rate, using a spectral feature-based detection method during a simulated driving task. However, they used a binary state to determine whether the subject was in Stage 1 sleep, rather than other drowsiness markers used in other work.

Dunne et al. (2015) investigated a real-time, Stage 1 sleep detection system. The EEG signal was filtered into low alpha, high alpha, high beta, and low beta bands and then used to predict potential sleep onset. The results suggested that even single-channel systems, such as the Neurosky MindWave, may be sufficient for real-time drowsiness detection schemes.

Joshi et al. (2015) performed a limited literature review describing EEG-based drowsiness and fatigue detection. Their review covered specific examples of the MindWave being used, due to its low cost. The low-cost EEG examples described in this review are also described here. However, they did not detail their search methodologies, nor did they include any EEG devices beside the MindWave.

Lin et al. (2015) used a Neurosky device as a real-time, EEG-based drowsiness detection device. The paper detailed a combined approach to drowsiness detection, integrating drowsiness detectors with other automobile safety features. However, no quantifiable results, such as accuracy, were summarized.

Putra et al. (2016) reported the development of an EEG-based microsleep detector for driving. The device used features from the different EEG spectral bands. However, no experimental results were reported in the paper.

Sadeghi et al. (2016) detailed the use of EEG-based drowsiness detection by passing data to wearable devices for processing. To scale the proposed SafeDrive app, the authors propose the HumaNet framework, which integrates both model-related and context-related information. The system was intended to work with both the Neurosky MindWave and the Emotiv Epov. The average performance values reported included an accuracy of 91%, a sensitivity of 83%, and a specificity of 99%.

Patel et al. (2017) evaluated the Neurosky MindWave specifically as a drowsiness detector. Using a driving simulator, a total of 7 subjects had EEG recorded. These 10 s sessions were divided into attentive driving and drowsy driving, and a paired *t*-test was performed on them. No statistically significant differences (*p* > 0.05) were found between averaged epochs for each category.

Anwar et al. (2018) used a Neurosky MindWave to record a meditating subject's EEG output and compared this with that of a 19-channel conventional EEG setup. Similar spectral changes were observed using both devices, although the measured amplitudes were different. Spectral data from different phases of meditation, such as the beginning and end, were also compared. Changes in the alpha and delta bands were noted.

Sethi et al. (2018) used a Mindwave device for assessing e-learning outcomes. EEG data was gathered from each subject (out of 42), without feedback. Following this, the subject was exposed to feedback for subsequent EEG recording sessions. Spectral features and proprietary parameters of attentiveness and meditation were compared for the same person, and then compared to the subject's EEG afterwards.

Aboalayon and Faezipour (2019) investigated a wireless EEG sleep stage detection system with a single channel Mindwave device. The system evaluated a real-time simulated driving task. However, the study was limited by its scope and length, precluding a definitive result on the device's performance.

Nissimagoudar and Nandi (2020) detailed an EEG detection system using alpha power, and using SVM for classification. The study used 10 subjects. The work detailed the expansion of a driver assistant, aimed at improving performance and safety behind the wheel. A range of classification results were reported from 74 to 89%, although highly dependent upon spatio-temporal features corresponding to drowsiness states.

Of those entries reporting accuracy, the performance ranges from 31 to 97.6%. As shown in Table 4, these results suggested that the Neurosky MindWave may be used for an EEG-based drowsiness detection system, although additional processing and feature extraction may be required.

**Table 4:** Neurosky MindWave Studies.

**Paper**	**Year**	**Features**	**Classification**	**Accuracy**	**Size**
Jones and Schwartz	2010	Spectral Features	N/A	N/A	5
AlZu'bi et al.	2013	PSD, log variance, stats	Fatigue index	N/A	1
Shin et al.	2013	Spectral Features	SVM	88.90%	1
Lim et al.	2014	Alpha band power	Triggering window	31%	50
Suprihadi and Karyono	2014	Spectral Features	Spectral threshold	68.11%	1
Abdel-Rahman et al.	2015	Periodogram	Neural network	97.60%	60
Dunne et al.	2015	Alpha and Beta Features	Threshold	81%	3
Joshi et al.	2015	Spectral Features	Threshold	N/A	1
Lin et al.	2015	Spectral Features	Threshold	N/A	1
Putra et al.	2016	Spectral Features	Threshold	N/A	0
Sadeghi et al.	2016	Alpha, Beta, Theta Power	Markov Chain Model	91%	1
Patel et al.	2017	Spectral Features	Paired *t*-test	N/A	7
Anwar et al.	2018	Spectral Features	Averaged threshold	75%	20
Sethi et al.	2018	Spectral Features, eSense	N/A	N/A	42
Aboalayon and Faezipour	2019	Spectral Features	N/A	N/A	1
Nissimagoudar and Nandi	2020	Spectral Features	SVM	74-89%	10

### OpenBCI Ultracortex

Karuppusamy and Kang (2017) used a 14-channel custom EEG headset with an OpenBCI board. They manually rated drowsiness periods using manually tagged videos of eye closure. The highest performing classifier reported was an SVM with a Gaussian kernel, with an accuracy of 81.2%.

Shen et al. (2017) reported a method of drowsiness detection beyond a binary state classifier. They did not report a specific accuracy, but they described a testing method that returned results that were independent of a subject's age and were based on the channels C4 and P3. They used a hybrid OpenBCI and Emotiv-based system to quantify spectral power across 50 test cases. According to the authors, the “depth of drowsiness” method described in this study was the first implementation of a non-binary drowsiness detector using a low-cost EEG system. They reported an accuracy of 82%, over a prior reported accuracy of 70% (Yin et al., 2011).

Mohamed et al. (2018a,b) used the Mark IV headset to analyze EEG output during driver behavior, based on spectral features. The input signal was divided into standard bands (delta, theta, alpha, and beta). To estimate the alertness level, the following feature extraction techniques were evaluated: the periodogram, Lomb-Scargle, multi-taper, and Welch's method. A multilayer neural network was used to evaluate the performance across all extracted features, with 10-fold cross-validation. The highest average classification accuracy was obtained using Welch's method, with 85.0% for testing accuracy. As the averaged sum of multiple periodograms, Welch's method was robust and not computationally intensive.

As shown in Table 5, these results suggested that the OpenBCI may be utilized for drowsiness detection, but the entire system must be assembled from component parts. Of the reported accuracies, the minimum was 79.4%, and the maximum was 96.4%. The additional complexity may decrease the accessibility relative to other systems.

**Table 5:** OpenBCI Studies.

**Paper**	**Year**	**Features**	**Classification**	**Accuracy**	**Size**
Karuppusamy and Kang	2017	PCA	Gaussian SVM	81.20%	N/A
Polosky et al.	2017	Spectral Features	Neural Network	N/A	1
Shen et al.	2017	Spectral Features	Threshold	82%	10
Mistry et al.	2018	Spectral Features	Threshold	79.40%	4
Mohamed et al.	2018	Spectral Features	Multilayer NN	96.40%	25

### Emotiv Insight and Epoc

Li and Chung (2014, 2015) used a combination of EEG and eyelid closure degree (ECD) to detect drowsiness. A smartphone was used as a processor, in conjunction with an Emotiv headset, resulting in a multimodal drowsiness detector. The phone's camera was used to detect ECD. The combined EEG-ECD detection system achieved an accuracy rate of up to 87.5%. They noted that the combination of the two measurements was able to overcome the shortcomings of each individual measurement.

Pomer-Escher et al. (2014) used spectral features from the alpha and theta bands of EEG. No real time classification was performed, but an ANOVA was conducted across features, channels, and conditions. In particular, the alpha power and ratio of theta to alpha were found to be measurements of fatigue.

Wang et al. (2015) proposed the use of sample entropy and rhythm energies for EEG-based mental fatigue estimation. A wavelet transform was used to find non-linear features in the EEG segment. Wavelet features and a backpropagation neural network (BPNN) were combined for classification. However, no accuracy was reported.

Dkhil et al. (2015, 2017) used an Epoc to validate a Fast Fourier Transform (FFT)-based method. A fuzzy logic system was used to assess drowsiness. This technique was also tested on Physionet sleep samples, but no accuracy value was reported.

Chen et al. (2016) compared four devices for drowsy driver detection: an Emotiv Epoc, a Neurosky MindWave, a camera, and a gyroscope. A total of three subjects were investigated. EEG spectral features were combined with regression for classification. The MindWave had an accuracy of 71%, but a high rate of misclassifications. The Emotiv Epoc had a reported accuracy of 92%, attributed to the greater number of electrodes. Compared with the other devices, EEG was found to be the most cost-effective means of driver detection.

Nugraha et al. (2016) and Sarno et al. (2016) used an Emotiv headset for drowsiness detection. Data from 30 volunteers were collected during driving simulator sessions that ranged from 33 to 60 min in length. A cross-channel correlation between spectral features was calculated for each subject. Both k-nearest neighbor (KNN) and SVM classifiers were used to detect drowsiness. The KNN system achieved a mean accuracy of 96%, whereas the SVM classifier achieved a mean accuracy of 81%.

Sawicki et al. (2016) examined a new measure for drowsiness detection, based on the maximum differences between the alpha band and the theta band, and a combined alpha-theta spectral power. An ANOVA was used to find significant differences between the feature under different lighting conditions. However, no accuracy value was reported.

Damit et al. (2017) developed a multi-modal fatigue estimation system for soldiers. The EEG of 10 subjects was gathered. Features extracted included spectral power and the discrete wavelet transform (DWT). In particular, the peak alpha frequency (PAF) was the primary EEG feature. No classification was performed, but a paired *t*-test was performed.

Alchalcabi et al. (2017) investigated the use of the Epoc+ as a tool for treating attention deficit hyperactivity disorder (ADHD) and attention deficit disorder (ADD). A virtual reality game was controlled using the headset. Instead of detecting drowsiness, the system was used to increase focus, and the authors reported an increase of 10% in healthy subjects that used the EEG-based controls.

Pham et al. (2018) directly examined real-time drowsiness detection using an Emotiv Epoc. The primary features extracted were spectral features, and classification was performed with SVM. The reported accuracy was 70%, with a single subject.

Poorna et al. (2018) investigated drowsiness detection in a driving simulation. Two feature sets were collected, spectral band powers and temporal characteristics. Principal component analysis (PCA) was used to reduce the number of features. Two algorithms were used for classification: k-nearest neighbor (KNN) and an artificial neural network (ANN). Reported classification accuracies were 80% for KNN and 85% for ANN.

Bajwa et al. (2019) tested a distracted driver detection system. A total of 13 subjects were investigated. Features include time-domain features and frequency-domain features, including spectral band power and wavelets. A multilayer perceptron (MLP) and Bayesian network were used for classification. Testing was performed while driving in a controlled an environment, an isolated parking lot. The reported accuracies include 91.54% for distraction detection, and 76.99% in identifying the cause of distraction.

Chen et al. (2019) explored the EEG of fatigue affecting drivers. Fourteen participants provided data for the study. EEG data was decomposed into band-based features using the wavelet packet transform (WPT). A parameter called phase-lag index (PLI) was proposed for network activity rating. Classification was performed with an SVM, and resulted in a reported accuracy of 94.4%.

Li et al. (2019) proposed four methods to identify fatigue. Most were derived from spectral features, which were used to establish a mental fatigue level (MFL). A simulated excavator task was used to validate the experiment with 15 participants. However, no classification was performed. However, an MF-based threshold was established from experimental data.

Rahma and Rahmatillah (2019) used an Epoc+ device for drowsiness data acquisition. EEG features were converted to discrete wavelet transforms (DWTs) and then subjected to common spatial patterns (CSP). The authors reported an average accuracy ranging from 91.67 to 93.75%, whereas the exclusion of CSP processing reduced the accuracy to no more than 87%.

Saichoo and Boonbrahm (2019) detailed a real-time driver drowsiness detection system used EEG band based spectral features, using the Emotiv Epoc+. Spectral band power was calculated using wavelets, Fourier transforms, and autoregressive estimates. Spatial filtering techniques, such as principal component analysis, were used for signal enhancement. Five volunteers were used. The system was able to correctly identify drowsiness at a rate of up to 83.33%, but had an overall accuracy of 70%. However, the system had difficulties with correct identification of non-drowsy states.

Tan et al. (2020) used data from a 40-min simulated driving task with 18 subjects, gathered with an Emotiv Epoc. Feature extraction involved band-based spectral power with a 2 s window. A time series classification (TSC) model was used, which assigned a label to each time segment. A Long-term Recurrent Convolutional Network (LCRN) was used for classification. As a preprint, the work still awaited peer review when discussed by the authors.

As shown in Table 6, the results suggest that the Emotiv Epoc and Epoc+ may be used for drowsiness detection, but proprietary firmware and software represent a potential issue. All entries reporting accuracy have a minimum of at least 80%. Support for Emotiv devices is difficult without the appropriate license.

**Table 6:** Emotiv Insight, Flex, and Epoc Studies.

**Paper**	**Year**	**Features**	**Classification**	**Accuracy**	**Size**
Li and Chung	2014–2015	Spectral, Eye Closure	SVM	82.71%	6
Pomer-Esche et al.	2014	Spectral Features	ANOVA	N/A	N/A
Dkhil et al.	2015–2017	Spectral Features	Fuzzy Logic Controller	N/A	1
Wang et al.	2015	Spectral, Wavelets, Entropy	BPNN	N/A	3
Chen et al.	2016	Spectral Features	Regression	92%	3
Nugraha et al.	2016	Spectral Features, Gyro	KNN, SVM	81–90%	6
Sawicki et al.	2016	Spectral Features	ANOVA	N/A	50
Alchalcabi et al.	2017	Spectral Features	State-based BCI	N/A	4
Damit et al.	2017	Wavelets, Spectral Features	Paired *t*-test	N/A	10
Pham et al.	2018	Spectral Features	SVM	70%	1
Poorna et al.	2018	Spectral Features	KNN, ANN	80–85%	18
Bajwa et al.	2019	Wavelets, Spectral Features	MLP, Bayesian Net	91.54%	13
Chen et al.	2019	PLI, Wavelet Transform	SVM	94.40%	14
Li et al.	2019	Spectral Features	MF Threshold	N/A	15
Rahma and Rahmatillah	2019	DWT	CSP	91.67–93.75%	1
Saichoo and Boonbrahm	2019	DWT, FT, AR	Thresholding	70%	5
Tan et al.	2020	Spectral Features	LCRN	83.33%	18

## Discussion

### Findings

Examples of EEG-based drowsiness detection were found for each examined brand, including the Emotiv Epoc, the Neurosky MindWave, the OpenBCI, and the InteraXon Muse, and all examined EEG systems were utilized in at least one successful example of real-time drowsiness detection (Abdel-Rahman et al., 2015; Nugraha et al., 2016; Mohamed et al., 2018b; Teo and Chia, 2018). A total of 27 surveyed studies reported an accuracy score. In terms of evaluated average accurate performance, the least consistent of these was the Neurosky MindWave, but the minimum of reported from the others was an OpenBCI study with 79.4% (Mistry et al., 2018). Although these systems may not be as accurate as research- or medical-grade systems, they may be sufficient for deployment in certain occupational contexts. For example, these systems could be deployed by smaller businesses in developing countries or by professions with the urgent need for easily available drowsiness detection systems. However, the different experimental designs utilized by each study make direct comparisons among these systems challenging.

Furthermore, several successful studies utilized relatively simple algorithms and spectral features, including FFT, EEG band powers, and linear classifiers (Abdel-Rahman et al., 2015; Nugraha et al., 2016; Mohamed et al., 2018b; Teo and Chia, 2018). The use of “complex” algorithms requiring more processing power, such as SVM, convolution neural networks, and deep learning systems, may constrain the ability to implement these systems (Nugraha et al., 2016). Even these more “complex” algorithms can easily run on an external device, such as a smartphone. Thus, even a single-electrode Neurosky Mindwave, when providing data to a properly trained classifier in controlled conditions, can achieve high accuracy in certain cases (Abdel-Rahman et al., 2015).

The usage of proven classification techniques and features demonstrates the relative ease of designing a drowsiness detection system, although low levels of accuracy, sensitivity, and specificity and the necessity of training are likely issues that may be encountered. However, the “best” headset depends on the user-specific trade-offs among algorithm complexity, performance, and price (AlZu'bi et al., 2013; Abdel-Rahman et al., 2015; Chen et al., 2016).

### Limitations

The current review had several limitations. First, the scope of the investigation was constrained by the small sample size. Second, the implementations and evaluation criteria greatly differed across the investigated papers, often using similar terminology for different concepts. For example, “drowsiness” was defined differently across studies, which included fatigue, microsleeps, and sleep stages (Vuckovic et al., 2002; Cabrall et al., 2016). In addition, the review included validation studies, conference results, and graduate dissertations, in addition to peer-reviewed journal articles. Future work would likely require alterations to the search and inclusion criteria.

### Future Work

Further steps are necessary to further examine the viability of using low-cost consumer EEG headsets as drowsiness detectors. First, the eligibility and search criteria should be further refined to more thoroughly cover the published literature. In addition, common performance metrics and definitions would ideally be described and consistently maintained. Comparisons of the data acquisition systems, feature extraction methods, and classification algorithms would be required. Finally, several additional brands and models of EEG headsets would need to be examined. Combining all of these steps would allow a more thorough meta-analysis to be performed.

## Conclusions

Traditional medical- and research-grade EEG systems have been successfully used for drowsiness and brain state estimation but are less versatile outside of a controlled laboratory environment. Between medical and consumer systems, innate limitations include a reduced number of electrodes, computation complexity, and noise removal capabilities. Low-cost EEG headsets show greater design convenience for “real world” occupational use. Several of these devices, including the Emotiv Epoc, Neurosky Mindwave, InterAxon Muse, and OpenBCI, have been utilized as drowsiness detectors, to varying degrees of success. However, open-source software and occupational refinement may boost the capabilities of these systems over time. This flexibility is advantageous to developing countries, small businesses, and hobbyist users; however, the final selection of optimal models and algorithms will be highly context-specific.

## Data Availability Statement

All datasets generated for this study are included in the article/supplementary material.

## Author Contributions

JL and ML performed the literature search. D-GP provided facilities and support. All authors contributed to the article and approved the submitted version.

## Conflict of Interest

The authors declare that the research was conducted in the absence of any commercial or financial relationships that could be construed as a potential conflict of interest.
